# EK-16A liposomes enhance HIV replication in ACH2 or J-Lat 10.6 cell engrafted NSG mice

**DOI:** 10.7150/ntno.69259

**Published:** 2022-03-21

**Authors:** Panpan Lu, Jinlong Yang, Xinyi Yang, Zhiming Liang, Jing Wang, Yanan Wang, Lin Zhao, Hanyu Pan, Xiaoting Shen, Yuqi Zhu, Jingna Xun, Hongzhou Lu, Huanzhang Zhu

**Affiliations:** 1 State Key Laboratory of Genetic Engineering and Engineering Research Center of Gene Technology, Ministry of Education, Institute of Genetics, School of Life Sciences, Fudan University, Shanghai, China.; 2 Department of Infectious Disease, Key Laboratory of Medical Molecular Virology of Ministry of Education/Health, School of Basic Medical Sciences and Shanghai Public Health Clinical Center, Fudan University, Shanghai, China.

**Keywords:** HIV, LRAs, *Euphorbia kansui*, EK-16A, NSG mouse model

## Abstract

**Background:** Numbers of HIV latency reversal agents (LRAs) have been tested in clinical trials, but with limited effect. EK-16A is an ingenol derivative that isolated from *Euphorbia kansui*. Our prior studies have suggested that it could reactivate latent HIV and meanwhile inhibit HIV infection *in vitro*. Here, we further advanced the research *in vivo*.

**Methods:** *In vitro*, the activity of EK-16A liposomes was measured in HIV latently infected cells. In serum pharmacology test, BALB/c mice were orally administered with EK-16A liposomes, serum was separated and co-cultured with cells, HIV reactivation was measured. *In vivo*, NSG mice were transplanted with human cells for 3 weeks and then administered with EK-16A liposomes for 3 days. In ACH2 cell engrafted NSG mice, P24 in plasma and cell-associated HIV RNA in tissues was measured. In J-Lat 10.6 cell engrafted NSG mice, GFP expression of J-Lat 10.6 cells in diverse tissues was measured. Hematoxylin and eosin (HE) staining was carried out for histopathological examination in both mice.

**Results:** EK-16A liposomes can reactivate latent HIV in ACH2 and J-Lat 10.6 cells. Serum pharmacological test showed that EK-16A retained activity after oral administration. Importantly, in ACH2 cell engrafted NSG mice, EK-16A liposomes increased the secretion of P24 in plasma and the expression of cell-associated HIV RNA in tissues. In J-Lat 10.6 cell engrafted NSG mice, EK-16A liposomes increased the GFP expression of J-Lat 10.6 cells in diverse tissues, including the bone marrow, spleen, liver, lung and peripheral blood. Furthermore, there was no obvious histopathological change associated with the use of EK-16A liposomes in both mice.

**Conclusions:** Our results confirmed the enhancing HIV replication activity and preliminary security of EK-16A in human cell engrafted NSG mice, laying the foundation for research in clinical trials.

## Introduction

Antiretroviral therapy (ART) effectively suppresses viral replication and partially restores immune functions in HIV infected individuals 1-3. However, ART cannot eliminate transcriptionally silent proviruses. A small pool of resting cells harbors latent replication competent HIV, and this latent reservoir remains untouched by current ART 4-6. It represents a key challenge in the eradication of HIV 7, 8. One potential strategy for eliminating the latently infected cells is termed as “shock and kill” in which LRAs are used to shock the latent virus out of the resting cells to allow host immune system, with the assistance of immunomodulatory therapies, to kill the virus ex pressing cells. This “shock and kill” procedure would typically be performed in those receiving ART to prevent additional rounds of viral replication 9.

A variety of compounds are under investigation as candidate LRAs for the shock step. Epigenetic LRAs targeted the chromatin restrictions that maintain HIV latency. These LRAs fall into three groups: histone deacetylase inhibitors 10-13, histone methyltransferase inhibitors 14 and bromo- and extra-terminal domain inhibitors 15-17. Signal Agonist LRAs stimulate immune signaling pathways in T cells, leading to transcriptional modifications and NF-κB activation. These LRAs include PKC agonists 18, cytokines 19-21, Toll-like receptor agonists 22-24, mimetics of the second mitochondrial-derived activator of caspases 25, and immune checkpoint inhibitors 26, 27. Clinical trials using SAHA 10, 28-31, romidepsin 13, 32, panobinostat 11, bryostatin-1 33, disulfiram 34 and so on showed some promise, but failed to result in significant reduction in the frequency of infected cells 19. It is urgent to develop more potent and safer LRAs.

*Euphorbia kansui* has been prescribed for thousands of years in traditional Chinese medicine. We previously reported that effective fractions from the dichloromethane extracts of the root of *Euphorbia kansui* can reactivate latent HIV replication in different latent cells (The 24th China science technology Forum-High level Forum on HIV cure, December 16-17, 2012, Beijing), and obtained the Chinese patent application approval and authorization (CN102727563B, CN106928063A). Recently, *Euphorbia kansui* has received increasing scientific attention for HIV latency reversal 35, 36, other researchers and we have launched clinical trials successively (clinicaltrials.gov, Identifier NCT02531295, NCT04503928). EK-16A is an ingenol derivative that extracted from *Euphorbia kansui* 37. Our prior studies have suggested that it could reactivate latent HIV by inducing both NF-κB and P-TEFb signaling pathways 38 and meanwhile inhibit HIV infection by down-regulating the expression of cell surface HIV co-receptors CCR5 and CXCR4 39 *in vitro*. In the current study, EK-16A was prepared into liposomes. The activity of EK-16A liposomes on enhancing HIV replication was evaluated *in vitro* and importantly in ACH2 and J-Lat 10.6 cell engrafted NSG mice *in vivo*.

## Methods

### Preparation of reagents

EK-16A is a chemical compound that isolated from *Euphorbia kansui* and the structure is as described in the previous study 38. In the current study, it was prepared as liposomes. Briefly, lecithin and cholesterol (Kewpie Corporation) were dissolved in dichloromethane, into which EK-16A solution (1 mg/mL) was added. The mixture formed a w/o emulsion upon sonication, which was subsequently evaporated under reduced pressure with a rotating speed of 50 rpm at 40 ℃ to remove the organic solvents. After that, PBS (pH 7.4) was added to hydrate the lipids until a homogeneous dispersion was formed. Finally, this dispersion was extruded though a high-pressure homogenizer to obtain liposomes 40. Vehicle control was prepared by the same procedure but EK-16A was not included in formulation. SAHA (Selleckchem) was suspended in sterile water plus 0.5% methylcellulose and 0.1% Tween 41.

### Cell culture

ACH2 cells are a CEM T-cell clone containing one integrated proviral copy of latent HIV-1 LAV 42, 43. J-Lat 10.6 cells are a Jurkat T-cell clone containing a full-length integrated HIV genome that expresses GFP upon activation 44. Both cell lines were obtained from NIH AIDS Reagent Program. They were cultured in RPMI1640 medium with 10% fetal bovine serum (FBS) and 1% Pen/strep in a 37 °C incubator containing 5% CO_2_.

### Ethics statement

All animal studies were reviewed and approved by Animal Ethic Committee of Fudan University.

### Measurement of HIV reactivation *in vitro*

ACH2 cells or J-Lat 10.6 cells were added at equal amounts for a total of 5×10^5^ cells per well to 24-well plates containing compound titrations. Dose-response testing was performed on EK-16A liposomes dissolved in PBS to give final assay concentrations ranging from 100 ng/mL to 0.16 ng/mL in 500 μL of medium. Cells and compound were incubated for 48 h. HIV reactivation in ACH2 cells was measured by the secretion of P24 antigen in supernatant using HIV Gag P24 ELISA kit (R&D Systems). HIV reactivation in J-Lat 10.6 cells was measured by the expression of GFP using flow cytometry (Beckman Coulter) 45. Data were analyzed using FlowJo software (V10).

### Serum pharmacological test

BALB/c mice aged 6 weeks were purchased from Shanghai SLAC Laboratory Animal Co., Ltd and randomized for assignment to either experimental or control groups. EK-16A liposomes were orally administered once daily at 0, 0.5 or 1mg/kg body weight for 3 days. Before drug administration, and after the last administration for 1 h, 2 h and 3 h, blood was collected from the retro-orbital venous sinus (r.o.). Serum was acquired by centrifugation the sample at 800 g for 10 min and then was calefied in 56 °C water for 30 min. Finally, 450 μL cells (5 × 10^5^) were co-cultured with 50 μL serum for 48 h and HIV induction was measured as *in vitro* 46-50.

### Cytotoxicity assay

This assay was performed as the protocol of the Cell Counting Kit-8 (CCK-8) (Dojindo Molecular Technologies). Briefly, cells were treated with EK-16A liposomes or serum from mice for 48 h. Then CCK-8 solution was added to each well. After 4 h of incubation at 37 °C, the absorbance at 450 nm was measured using a microplate reader (BioTec) 38.

### Cell surface marker staining

Cells were washed once with PBS, resuspended in 100 µL PBS, stained with human CD147-APC (BioLegend), CD29-APC (BioLegend), HLA-ABC-APC (BD), CD3-PE (BD), CD4-PE (Miltenyi) or TCR α β-FITC (BD) antibodies, respectively, and were incubated for 30 min. Cells were then washed with 1 mL of PBS, resuspended in 500 µL PBS, and measured by flow cytometry 51.

### Establishment and analysis of cell engrafted NSG mice

NSG mice (NOD-*Prkdc^scid^Il2rg^em1^/Smoc*, NM-NSG-001) 52-54 aged 6 weeks were purchased from Shanghai Model Organisms. NSG mice were transplanted with 1 × 10^7^ ACH2 cells or J-Lat 10.6 cells in a volume of 200 µL PBS by tail vein injection. Cell engrafted mice as well as control mice were sacrificed by cervical dislocation at the indicated time. Bone marrow, spleen, liver, lung, and peripheral blood were harvested. Single cell suspensions from these tissues were prepared as described previously 51, 55. For the measurement of engraftment, cells were stained with human CD147-APC and measured by flow cytometry 51.

### Treatment of cell engrafted NSG mice

NSG mice were transplanted with ACH2 cells or J-Lat 10.6 cells. Three weeks post-transplantation, they were randomized for assignment to either experimental or control groups. Cell engrafted mice respectively received 3 times of EK-16A liposomes (1 mg/kg), SAHA (60 mg/kg) or vehicle control orally. They were euthanized 24 h after the last dose.

### Measurement of HIV reactivation *in vivo*

In ACH2 cell engrafted mice, HIV induction was analyzed by monitoring the change of plasma P24 antigen before and after treatment. The level of P24 antigen in plasma was determined using HIV Gag P24 ELISA kit according to the manufacturer's protocol. HIV induction was also analyzed by comparing tissue HIV RNA of LRA treated mice versus the vehicle control 25. Single cell suspensions from tissues were prepared. RNA was extracted using ZR-96 Viral RNA Kit (ZYMO Research). cDNA was synthesized using BeyoRT III cDNA kit (Beyotime) and analysed using QuantiFast SYBR Green PCR Kit (Qiagen) on a Roche LightCycler 480 II machine. HIV *gag* expression was normalized to *GAPDH* and the comparative threshold cycle (Ct) method (ΔΔCt) was used for relative quantification of gene expression. Results were presented as fold induction relative to vehicle control. HIV viral RNA levels in cells isolated from the bone marrow, spleen, liver, lung and peripheral blood of control or LRA-treated mice (cells pooled from n = 5 mice per group for each tissue) were analyzed in triplicate. The primer sequences of HIV *gag* detection were: 5′- ACATCAAGCAGCCATGCAAAT-3′, 5′-TCTGGCCTGGTGCAATAGG-3′. The primer sequences of *GAPDH* detection were: 5′-TCAAGTGGGGCGATGCTGGC-3′, 5′-TGGGGGCATCAGCAGAGGGG-3′ 56.

In J-Lat 10.6 cell engrafted mice, HIV induction was analyzed by comparing GFP expression of J-Lat 10.6 cells in tissues of LRAs treated mice versus the vehicle control group. GFP expression of engrafted J-Lat 10.6 cells was determined by flow cytometry 51.

### Histopathological examination

Cell engrafted NSG mice were treated with LRAs. At 24 h after the last dose, mice were sacrificed. Heart, liver, spleen, lung and kidney were collected and fixed in formalin overnight. Tissues were then processed and sectioned for HE staining as described previously 57.

### Statistical analysis

All data were analyzed using GraphPad Prism Software (V6) and are presented as means ± the standard error of the mean (SEM). Applied statistical tests and analyses are described in figure legends. P ≤ 0.05 was considered statistically significant. At least three samples were used for each group, the minimum to achieve statistical significance.

## Results

### EK-16A liposomes reactivate latent HIV *in vitro*

**Figure 1:**
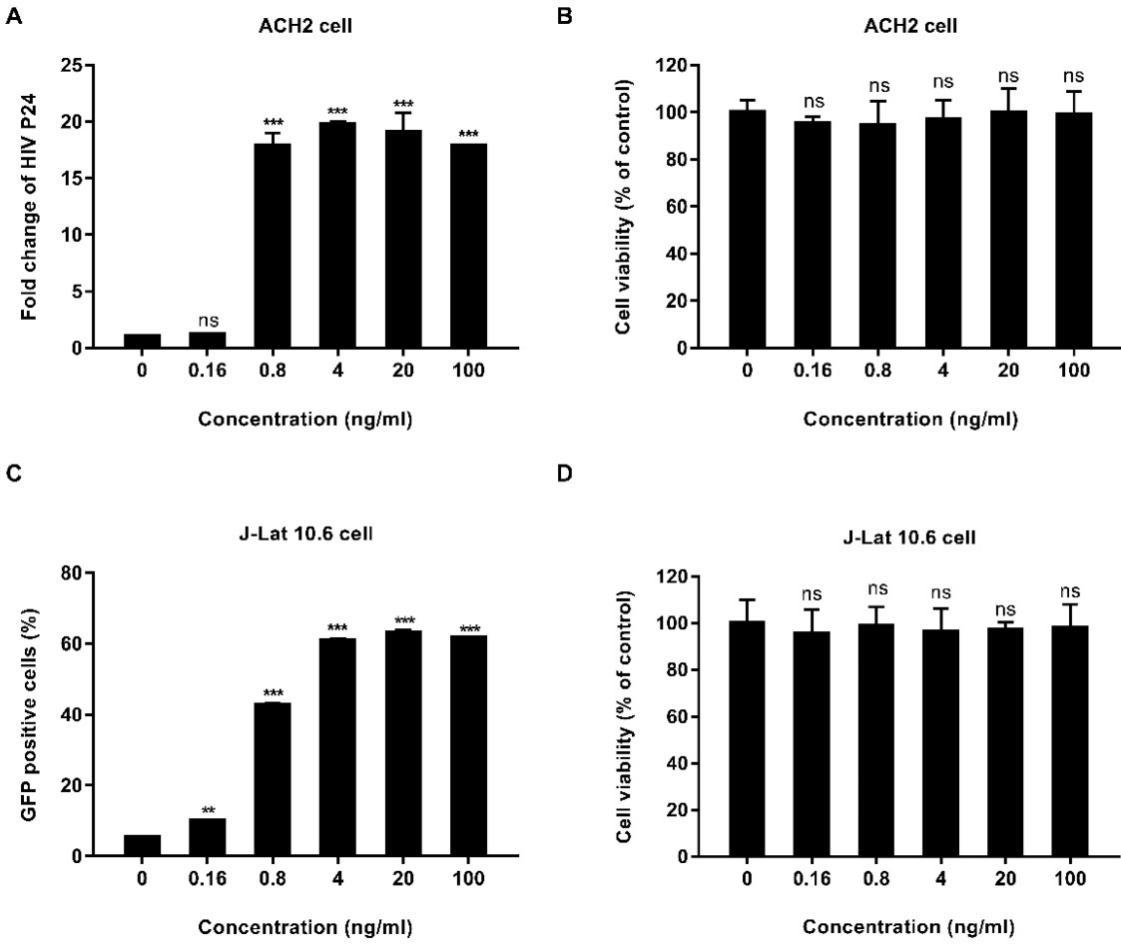
** Latent HIV reactivation by EK-16A liposomes *in vitro*.** ACH2 or J-Lat 10.6 cells were treated with EK-16A liposomes at the indicated concentrations. (A) HIV expression in ACH2 cells was measured by quantifying the level of p24 in the supernatant and presented as fold induction relative to control. (B) The viability of ACH2 cells was measured using CCK-8 kit. The division of OD450 between treated and control groups indicate the percentage of cell viability. (C) HIV expression in J-Lat 10.6 cells was measured by quantifying the percentage of GFP-positive cells. (D) The viability of J-Lat 10.6 cells was measured as in (C). Data show the means ± SEM. Statistical significance was determined using a student t test, ns *p* > 0.05, * *p* ≤ 0.05, ** *p* ≤ 0.01, *** *p* ≤ 0.001.

We tested the activity of EK-16A liposomes *in vitro*. ACH2 cells were treated with EK-16A liposomes for 48 h, the level of P24 antigen in the supernatant was detected by ELISA. Results showed that when the concentration of EK-16A increased from 0 to 4 ng/mL, the level of P24 antigen continued to increase. When the concentration of EK-16A reached 4 ng/ml, the level of P24 antigen reached the plateau, which was 19.74-fold higher than control. EK-16A liposomes induced HIV expression in a dose-dependent manner in the absence of any apparent cellular toxicity in ACH2 cells (Figure 1A and 1B).

The activity of EK-16A liposomes was also verified in J-Lat 10.6 cells. J-Lat 10.6 cells were treated with EK-16A liposomes for 48 h, the proportion of GFP-positive cells was detected by flow cytometry. Results showed that when the concentration of EK-16A liposomes increased from 0 ng/mL to 20 ng/mL, the proportion of GFP-positive cells increased from 5.45% to 63.03%. When the concentration of EK-16A reached 20 ng/ml, the proportion of GFP-positive cells reached the plateau. EK-16A liposomes induced HIV expression in a dose-dependent manner with no effect on cell viability in J-Lat 10.6 cells (Figure 1C and 1D).

### Serum from BALB/c mice receiving EK-16A liposomes reactivate latent HIV

**Figure 2:**
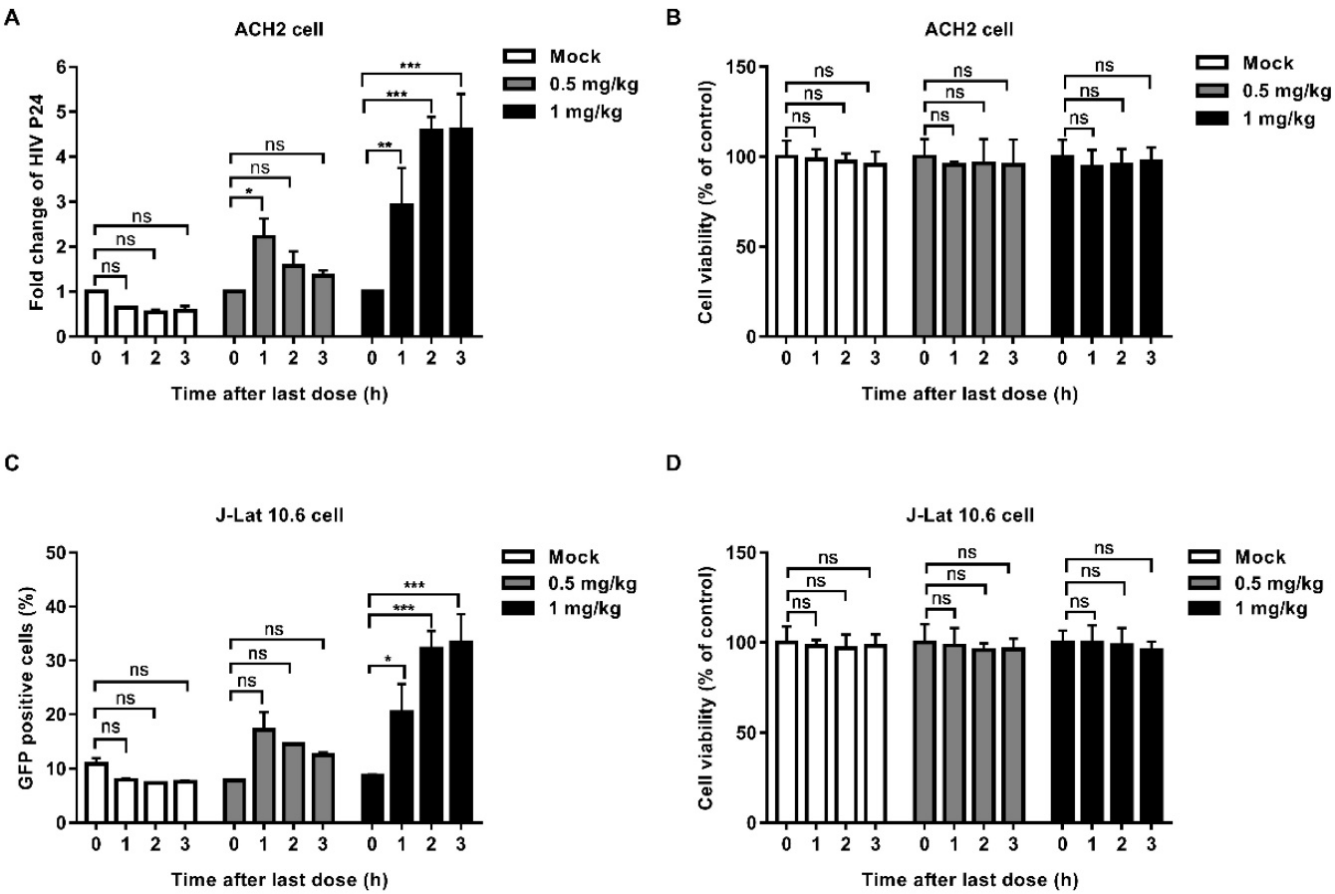
** Latent HIV reactivation by serum from BALB/c mice receiving EK-16A liposomes.** EK-16A liposomes were administered to BALB/c mice at the dose of 0, 0.5 or 1 mg/kg for 3 days. Before treatment (0 h) and after the last administration for 1 h, 2 h and 3 h, serum was separated and co-cultured with ACH2 or J-Lat 10.6 cells for 48 h. (A and B) In ACH2 cells, the level of p24 antigen in the supernatant (A) and cell viability (B) was analyzed. (C and D) In J-Lat 10.6 cells, the percentage of GFP-positive cells (C) and cell viability (D) was analyzed. N=3, data show the means ± SEM. Statistical significance was determined using a two-way ANOVA test, ns *p* > 0.05, * *p* ≤ 0.05, ** *p* ≤ 0.01, *** *p* ≤ 0.001.

In order to explore whether EK-16A can be absorbed into blood and retain activity after oral administration, we performed the serum pharmacological test. EK-16A liposomes were given to mice orally once a day for 3 consecutive days. Blood was collected before treatment and after the last administration. Serum was separated and co-cultured with latently infected cells for 48 h to test whether it can promote the expression of HIV. In the supernatant of ACH2 cells, the level of P24 antigen induced by serum collected from mice receiving 1 mg kg^-1^ EK-16A treatment at 1 h, 2 h and 3 h after the last administration was respectively 2.92-fold, 4.57-fold and 4.61-fold higher than control. The level of P24 antigen induced by serum collected from mice receiving 0.5 mg/kg EK-16A treatment at 1 h after the last administration was 2.22-fold higher than control (Figure 2A). On J-Lat 10.6 cells, the proportion of GFP-positive cells induced by serum collected from mice receiving 1 mg/kg EK-16A liposomes treatment at 1 h, 2 h and 3 h after the last administration was respectively 20.4%, 32.2% and 33.4%, significantly higher than control, which was 8.77% (Figure 2C). Compared to serum collected before treatment, serum collected after treatment made no apparent cellular toxicity in ACH2 cells (Figure 2B) and J-Lat 10.6 cells (Figure 2D).

### ACH2 and J-Lat 10.6 cell engrafted NSG mouse models are established

**Figure 3:**
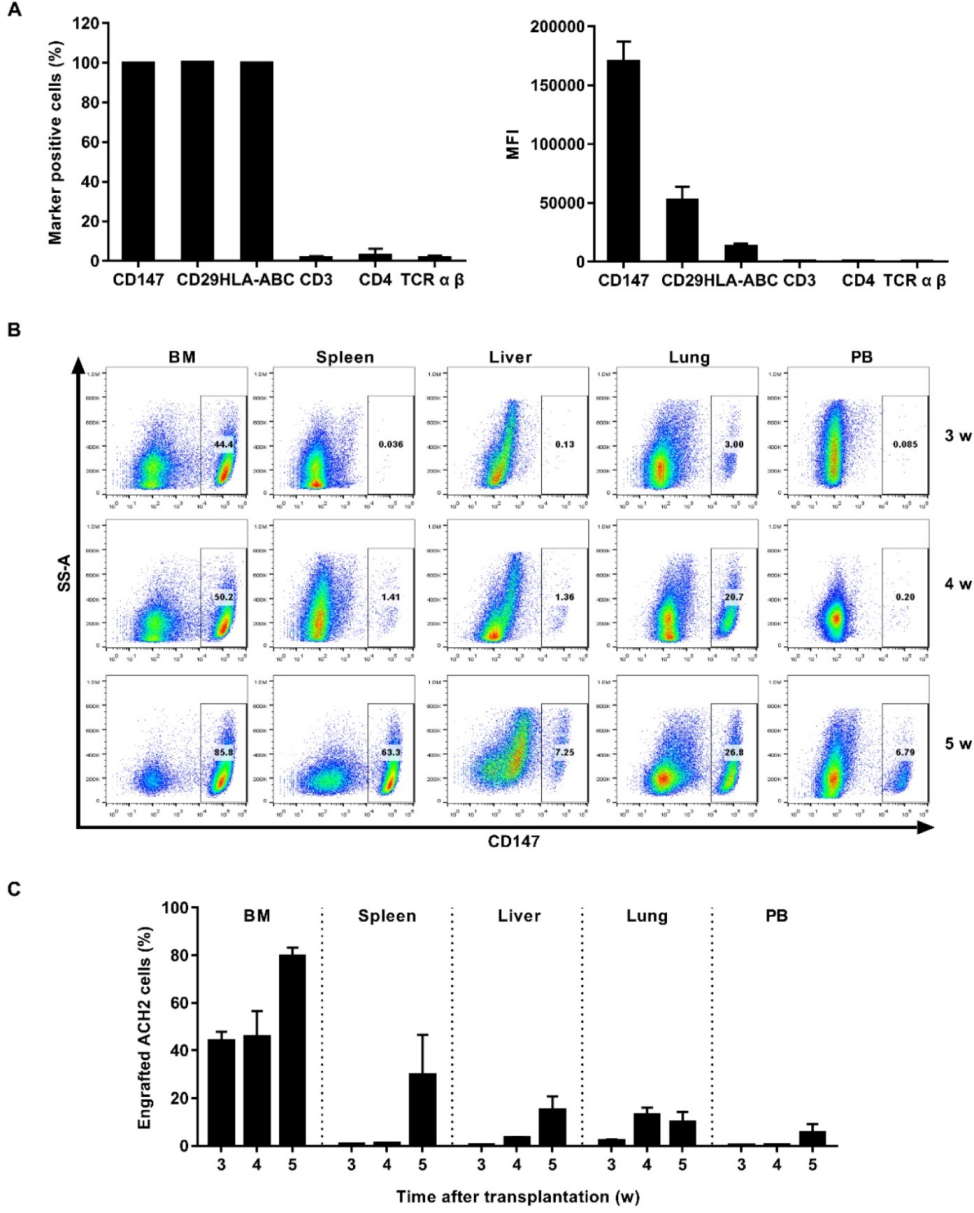
** Establishment of ACH2 cell engrafted NSG mouse model.** (A) ACH2 cells were stained with antibodies targeting select human cell surface, the frequency of marker-positive cells and MFI was measured by flow cytometry. (B) NSG mice were engrafted with ACH2 cells for 3, 4 or 5 weeks, respectively. Representative flow cytometry plots were shown for the engraftment level in tissues. (C) Bar graphs summarized the frequency of engrafted ACH2 cells in respective tissue. N=5, data show the means ± SEM.

ACH2 cell engrafted NSG mouse model was used for *in vivo* studies. We first searched for cell surface marker that identified engrafted ACH2 cells in a background of mouse cells. Our candidate panel included six cell surface proteins commonly known to be expressed in human CD4^+^ T cells: CD147, CD29, HLA-ABC, CD3, CD4 and TCR α β. Three of these cell surface proteins were found to be expressed universally among the ACH2 population: CD147, CD29 and HLA-ABC. The mean fluorescence intensity (MFI) of the proteins, reflecting the relative abundance of each protein on the cell surface, was measured. CD147 exhibited the highest MFI (Figure 3A). We next tested if the CD147 antibodies showed detectable binding to NSG mouse cells obtained from different tissues. While 100% of cultured ACH2 cells expressed (Figure S1A), the frequency of CD147^+^ cells was negligible or absent across mouse tissues (Figure S1C). Thus, human CD147 could function as a specific marker for the identification of ACH2 cells engrafted in NSG mice.

NSG mice were engrafted intravenously with 1×10^7^ ACH2 cells. The engraftment of ACH2 cells in different tissues was monitored by flow cytometry every week. Results showed that ACH2 cells appeared in the main immune tissues in 3 weeks, and reached the peak in 5 weeks (Figure 3B). The mean frequency of engrafted ACH2 cells varied across tissues. Three weeks post transplantation, it was approximately 43.86% in bone marrow, 0.72% in spleen, 0.40% in liver, 2.17% in lung and 0.22% in peripheral blood. Five weeks post transplantation, it was approximately 79.62% in bone marrow, 29.74% in spleen, 15.03% in liver, 10.04% in lung and 5.51% in peripheral blood (Figure 3C). Notably, starting from the 4th week after ACH2 cell transplantation, weights of mice fluctuated significantly (Figure S2A). Considering both transplantation efficiency and body weight, this model is best used in 3 weeks.

**Figure 4:**
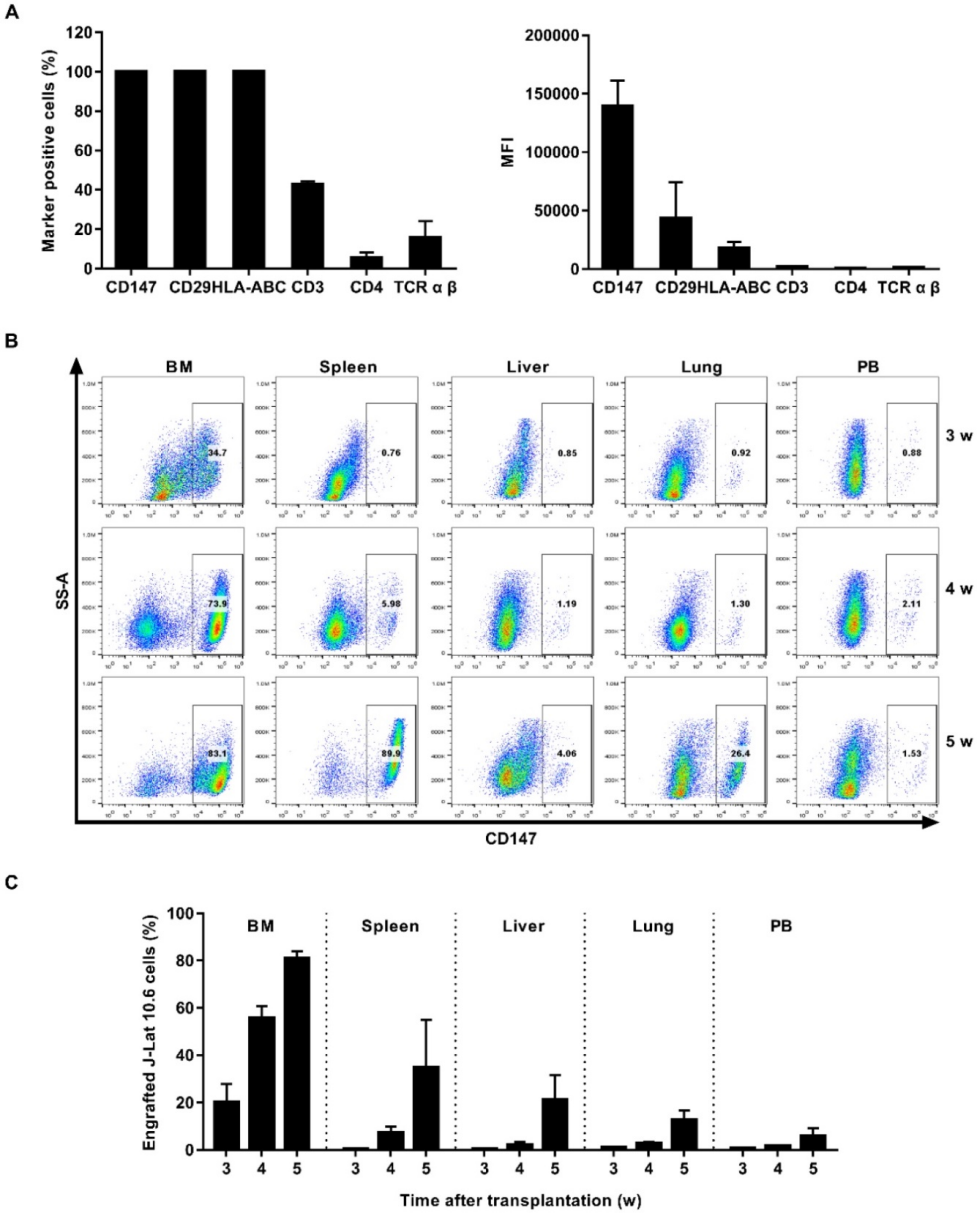
** Establishment of J-Lat 10.6 cell engrafted NSG mouse model.** (A) J-Lat 10.6 cells were stained with antibodies targeting select human cell surface, the frequency of marker-positive cells and MFI were measured by flow cytometry. (B) NSG mice were engrafted with J-Lat 10.6 cells for 3, 4 or 5 weeks, respectively. Representative flow cytometry plots were shown for the engraftment level in tissues. (C) Bar graphs summarized the frequency of engrafted J-Lat 10.6 cells in respective tissue. N=5, data show the means ± SEM.

J-Lat 10.6 cell engrafted NSG mouse model was also used. CD147 protein was expressed universally and exhibited the highest MFI among the J-Lat 10.6 population (Figure 4A and Figure S1B). NSG mice were engrafted intravenously with 1×10^7^ J-Lat 10.6 cells. J-Lat 10.6 cells appeared in the main immune tissues in 3 weeks, and reached the peak in 5 weeks (Figure 4B). Three weeks post transplantation, it was approximately 20.32% in bone marrow, 0.55% in spleen, 0.53% in liver, 1.06% in lung and 0.59% in peripheral blood. Five weeks post transplantation, it was approximately 80.86% in bone marrow, 34.84% in spleen, 21.30% in liver, 12.80% in lung and 5.91% in peripheral blood (Figure 4C). Starting from the 4th week after J-Lat 10.6 cell transplantation, weights of mice fluctuated significantly (Figure S2B). Overall, this model is best used in 3 weeks.

### EK-16A liposomes enhance HIV replication in cell engrafted NSG mouse models

**Figure 5:**
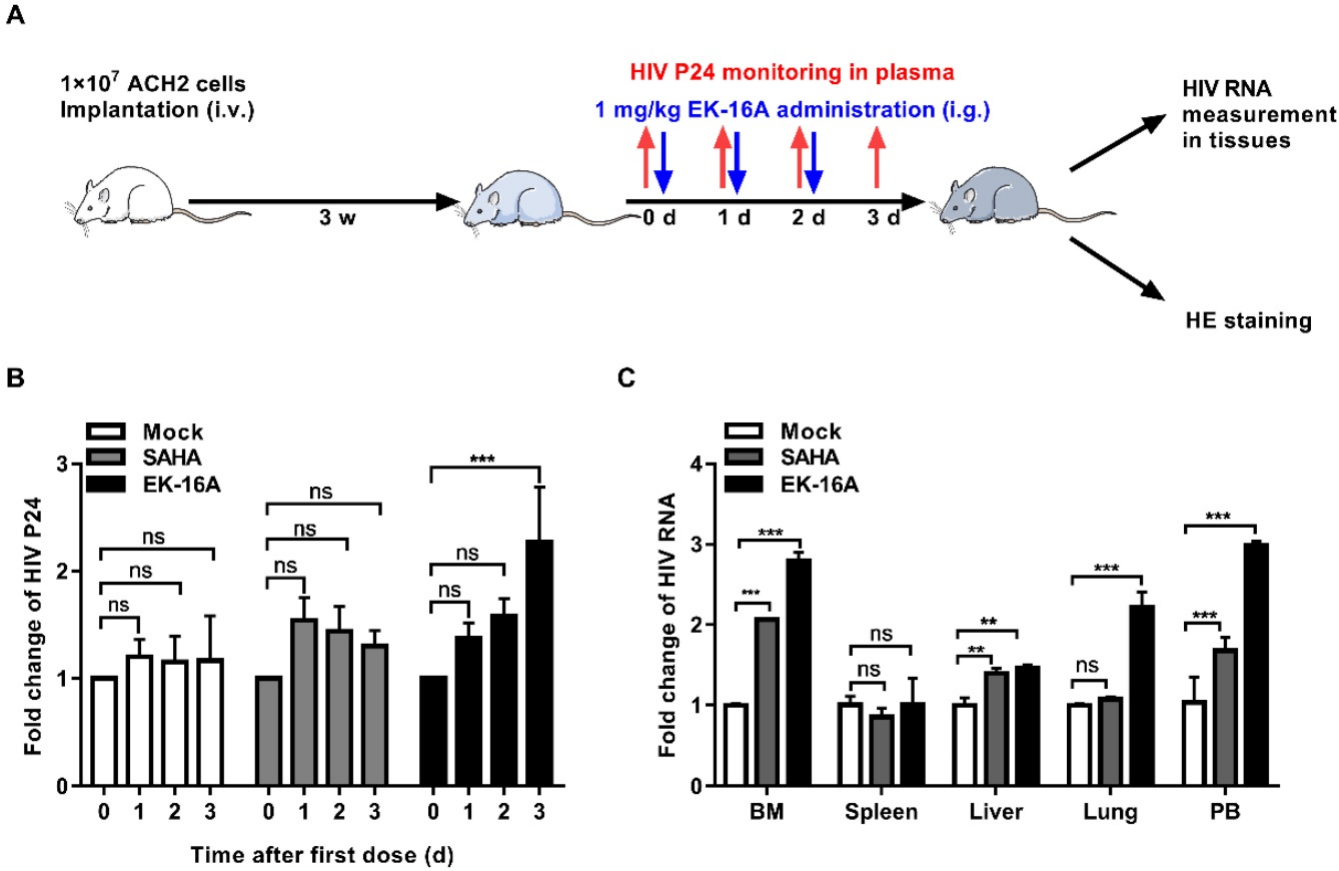
** Enhancing HIV replication by EK-16A liposomes in ACH2 cell engrafted NSG mouse model.** (A) Experimental design during EK-16A liposomes treatment phase in ACH2 cell engrafted NSG mice. (B) NSG mouse models were treated with vehicle control, SAHA or EK-16A liposomes for 3 days. The level of P24 in plasma was detected every day and results were presented as fold induction relative to prior treatment for each mouse. (C) After treatment, HIV RNA from cells of respective tissue were analyzed and results were presented as fold induction relative to control. N=5, data show the means ± SEM. Statistical significance was determined using a two-way ANOVA test, ns *p* > 0.05, * *p* ≤ 0.05, ** *p* ≤ 0.01, *** *p* ≤ 0.001.

ACH2 cell engrafted NSG mice were divided into 3 groups and received oral treatment with 1 mg/kg EK-16A liposomes, 60 mg/kg SAHA or vehicle control for 3 days, respectively (Figure 5A). After 3 consecutive EK-16A liposomes treatment, increased P24 antigen secretion was detected in the plasma of mice. However, no change in P24 antigen level was detected in vehicle-control-treated mice nor in SAHA-treated mice (Figure 5B). The hallmark of HIV persistence in humans is the presence of inducible HIV in different tissues. We isolated cells from primary (bone marrow), secondary (spleen), effector (liver and lung) immune tissues and peripheral blood after treatment. The level of HIV RNA from EK-16A-treated mice was significantly higher in bone marrow (2.80-fold), liver (1.46-fold), lung (2.22-fold) and peripheral blood (2.99-fold) compared with vehicle controls, but not in spleen. The level of HIV RNA from SAHA-treated mice was significantly higher in bone marrow (2.06-fold), liver (1.40-fold) and peripheral blood (1.68-fold) compared with vehicle controls, but not in spleen or lung (Figure 5C). Furthermore, no notable difference in histopathological examination was noted among ACH2 cell engrafted NSG mice treated with EK-16A liposomes, SAHA or vehicle control (Figure 7A). Overall, results demonstrate that EK-16A liposomes induce viral reactivation in ACH2 cell engrafted NSG mice.

**Figure 6:**
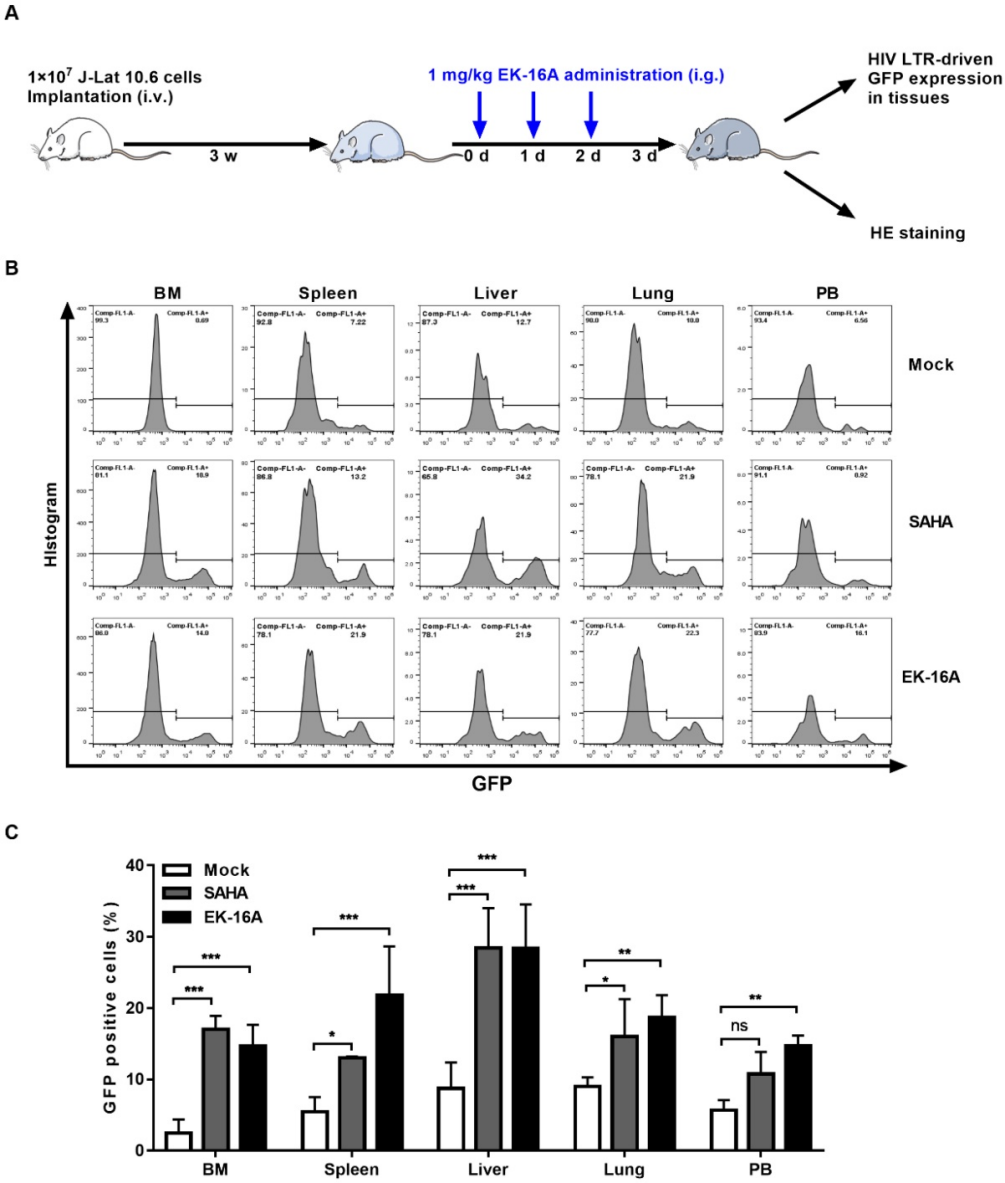
** Enhancing HIV replication by EK-16A liposomes in J-Lat 10.6 cell engrafted NSG mouse model.** (A) Experimental design during EK-16A liposomes treatment phase in J-Lat 10.6 cell engrafted NSG mouse model. (B) NSG mouse models were treated with vehicle control, SAHA or EK-16A liposomes for 3 days. Representative flow cytometry histograms were shown for the percentage of GFP positive cells within J-Lat 10.6 cells from respective tissue. (C) Bar graphs summarized the percentage of GFP positive cells within J-Lat 10.6 cells from respective tissue. N=5, data show the means ± SEM. Statistical significance was determined using a two-way ANOVA test, ns *p* > 0.05, * *p* ≤ 0.05, ** *p* ≤ 0.01, *** *p* ≤ 0.001.

**Figure 7:**
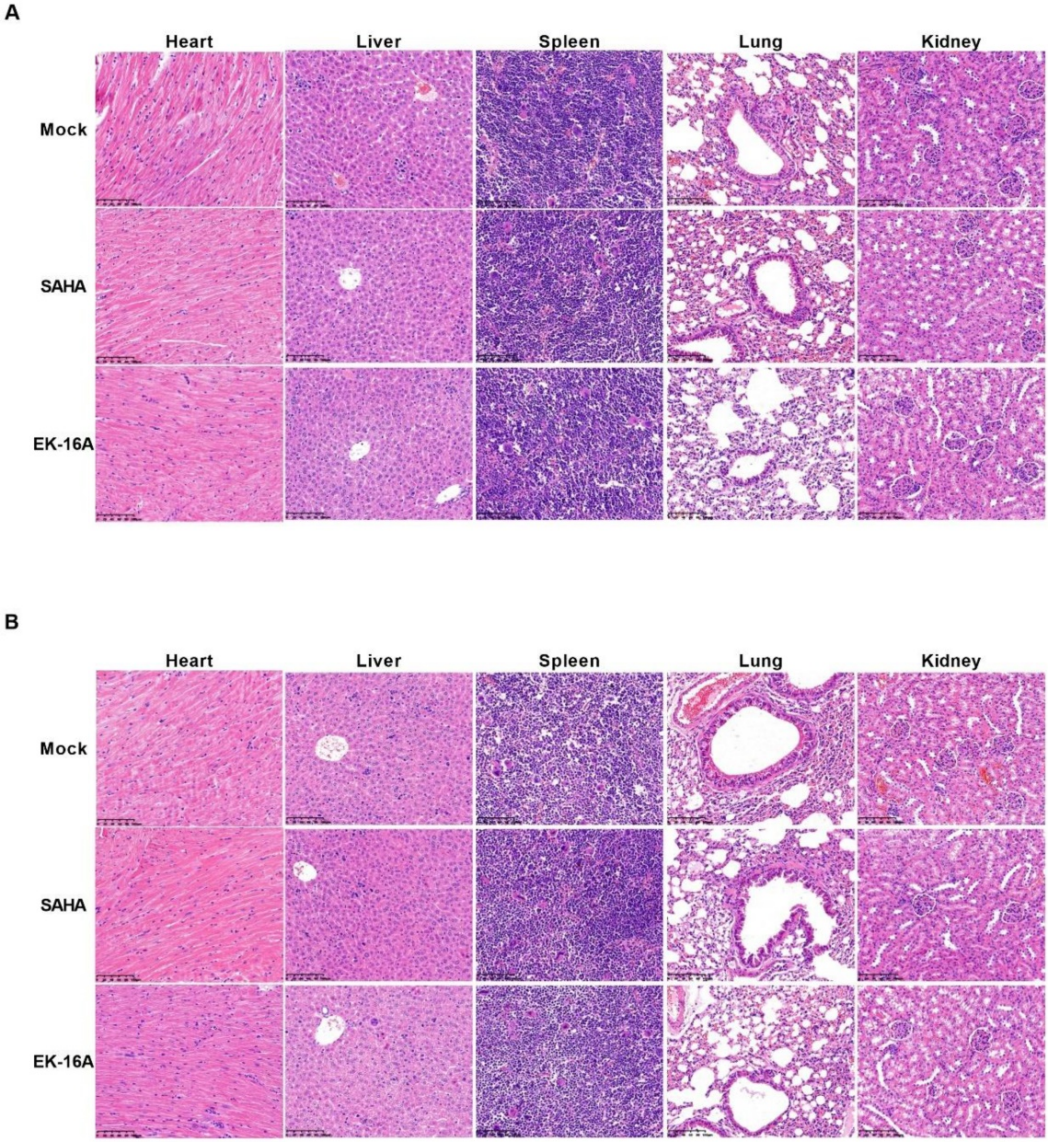
** Histopathological examination of NSG mouse models after EK-16A liposomes treatment.** After treated with vehicle control, SAHA or EK-16A liposomes for 3 days, ACH2 cell engrafted NSG mice (A) or J-Lat 10.6 cell engrafted NSG mice (B) were sacrificed. Heart, liver, spleen, lung and kidney were collected and HE staining was performed.

We also evaluated the activity of EK-16A liposomes in NSG mice 3 weeks post J-Lat 10.6 cell transplantation (Figure 6A). Viral reactivation was measured based on the frequency of GFP expressing cells within engrafted J-Lat 10.6 cells from mouse tissues following 3 consecutive treatment. EK-16A liposomes treatment did lead to increase in the frequency of GFP-positive cells, so did SAHA (Figure 6B). Statistics showed that the percentage of GFP-positive cells from EK-16A-treated mice was significantly higher in bone marrow (6.00-fold), spleen (4.00-fold), liver (3.24-fold), lung (2.08-fold) and peripheral blood (2.61-fold) compared with vehicle control. The percentage of GFP-positive cells from SAHA-treated mice was also significantly higher in diverse tissues compared with vehicle control (Figure 6C). There was no significant difference in histopathological examination of mice after treatment (Figure 7B). Taken together, results demonstrate that EK-16A liposomes induce viral reactivation in J-Lat 10.6 cell engrafted NSG mice.

## Discussion

Eradication of HIV infection after prolonged viral suppression is the focus of intense research and latency reversal has been a cornerstone of this effort. LRAs have been widely recognized as important tools to induce HIV expression. Future clinical applications of HIV cure strategies must be relevant to the majority of people living with HIV for whom the treatments of the London and Berlin patients 58, 59 pose an unacceptable level of risk. Therefore, LRAs must be identified that are highly effective but have minimal side effects 25.

EK-16A is an ingenol derivative that extracted from *Euphorbia kansui* 37. Our prior studies have suggested that it could reactivate latent HIV by inducing both NF-κB and P-TEFb signaling pathways 38 and meanwhile inhibit HIV infection by down-regulating the expression of cell surface HIV co-receptors CCR5 and CXCR4 39 *in vitro*. Here, we further advanced the research of EK-16A. EK-16A is a kind of oily compound with high hydrophobicity and low solubility. It was prepared into liposomes with lecithin and cholesterol to promote oral administration 60, 61. Two different systems, ACH2 and J-Lat 10.6, were used in the study. The concordance between the results obtained in two systems highlights the reproducible nature of the effect of EK-16A on enhancing HIV replication.

*In vitro*, EK-16A liposomes promote the secretion of P24 in ACH2 cells and the expression of GFP in J-Lat 10.6 cells, both with a dose-dependent effect. Serum pharmacological test, commonly used in traditional Chinese medicine 46-50, was introduced to prove that EK-16A were still active after being absorbed into blood. When serum was added into cell culture medium, the concentration of EK-16A was diluted 10 times, which resulted in less effective at some low doses.

*In vivo*, by applying widely utilized HIV latently infected cell lines, we achieved the establishment of two mouse models in short time, circumventing the need for donor derived tissues. ACH2 and J-Lat 10.6 cell engrafted NSG mouse models were scalable, accessible, and cost-effective. Although not intended to serve as pathophysiology models, they could be used to evaluate the performance of HIV cure strategies in distinct anatomical niches 51. SAHA, also known as vorinostat, a histone deacetylase inhibitor, has been widely evaluated as an LRA 28, 29, 31, was served as a control. In ACH2 cell engrafted NSG mice, multiple doses of EK-16A liposomes increased the secretion of P24 antigen in plasma and the expression of cell-associated HIV RNA in diverse tissues. The index of P24 antigen is not as sensitive as that of plasma viral RNA, but it is more convincing 56. In J-Lat 10.6 cell engrafted NSG mice, multiple doses of EK-16A liposomes increased the expression of GFP in J-Lat 10.6 cells from diverse tissues. Previous attempts to reactivate HIV in preclinical animal models and in clinical trials have measured HIV induction in the peripheral blood with minimal focus on tissue reservoir and have had limited effect 25. Our results provided *in vivo* evidence of systemic enhancing HIV replication for EK-16A liposomes. Importantly, there was no obvious histopathological change associated with EK-16A liposomes in the two mouse models. Of course, the safety of EK-16A liposomes needs much more stringent assessment in the future. For example, long-term toxicity experiments should be performed to exclude the possibility of accumulative toxicity in mice due in an extended time duration.

In summary, our results demonstrated the activity and preliminary security of EK-16A liposomes on enhancing HIV replication in human cell engrafted NSG mice, laying the foundation for research in more advanced animal models and clinical trials. The study of EK-16A liposome *in vivo* indicate that ingenol compound can be possibly tested in a more clinically relevant model of HIV in an appropriate formula. Undeniably, there were still some limitations. The study lacked dedicated “kill” interventions. Combining “shock” interventions with “kill” components is a key next step 62. This promising compound, in combination with appropriate tools for clearance of HIV infection, may increase opportunities for HIV eradication.

## Supplementary Material

Supplementary figures.

## Abbreviations

ART: antiretroviral therapy
BM: bone marrow
ELISA: enzyme linked immunosorbent assay
GFP: green fluorescent protein
HIV: human immunodeficiency virus
LRAs: latency reversal agents
LTR: long terminal repeat
MFI: mean fluorescence intensity
NF-κB: nuclear factor κB
PB: peripheral blood
PBS: phosphate buffer saline
PCR: phosphate buffer saline
PKC: protein kinase C
P-TEFb: positive transcription elongation factor b
SAHA: suberoylanilide hydroxamic acid
