# Supplementary material for: EK-16A liposomes enhance HIV replication in ACH2 or J-Lat 10.6 cell engrafted NSG mice
Source: Nanotheranostics. 2022 Mar 21;6(3):325–36. doi: 10.7150/ntno.69259 (PMC9194586; doi:10.7150/ntno.69259)
Supplement: Supplementary file 1 — Supplementary figures. [file ntnov06p0325s1.pdf]

Supplementary material for

EK-16A liposomes enhance HIV replication in ACH2 or J-Lat 10.6 cell engrafted NSG mice

Panpan Lu[1], Jinlong Yang[1], Xinyi Yang[1], Zhiming Liang[1], Jing Wang[1], Yanan Wang[1], Lin Zhao[1], Hanyu Pan[1], Xiaoting Shen[1], Yuqi Zhu[1], Jingna Xun[1,2], Hongzhou Lu[2], Huanzhang Zhu[1, *]

1. State Key Laboratory of Genetic Engineering and Engineering Research Center of Gene Technology, Ministry of Education, Institute of Genetics, School of Life Sciences, Fudan University, Shanghai, China.

2. Department of Infectious Disease, Key Laboratory of Medical Molecular Virology of Ministry of Education/Health, School of Basic Medical Sciences and Shanghai Public Health Clinical Center, Fudan University, Shanghai, China.

* Corresponding author: Huanzhang Zhu. E-mail: hzzhu@fudan.edu.cn

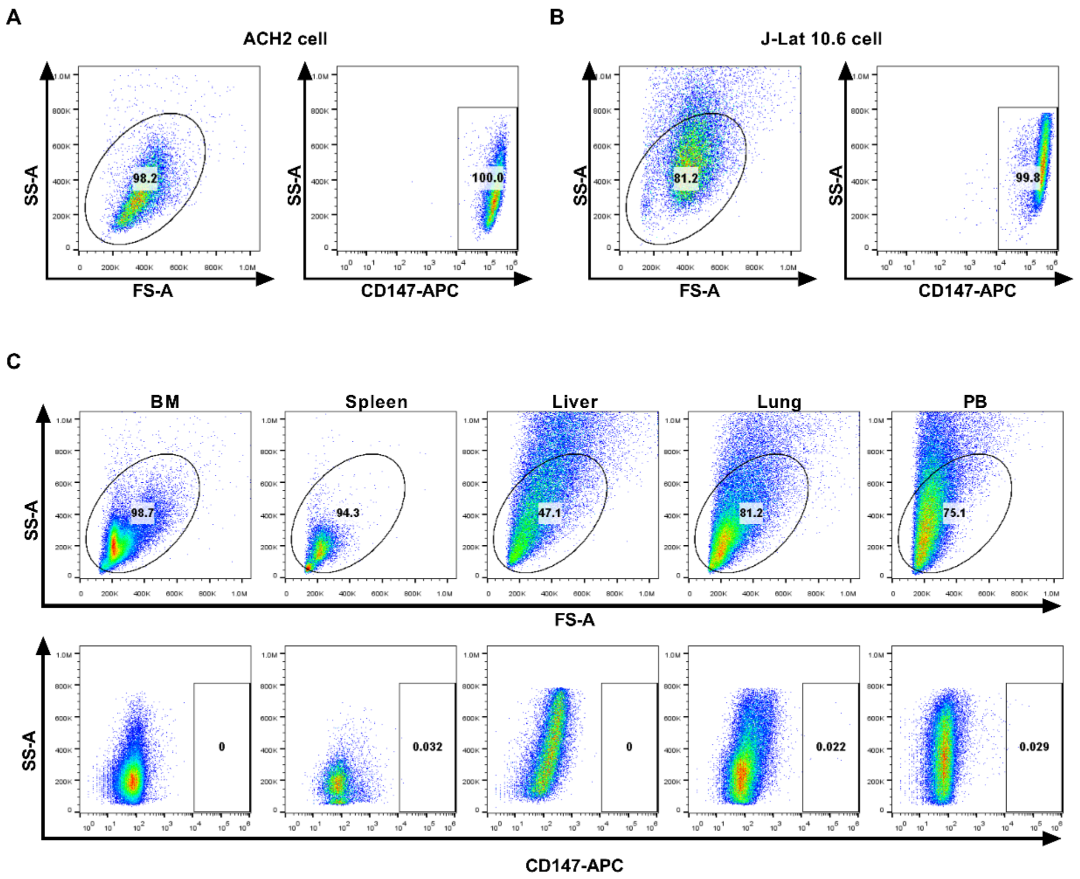

Supplementary Figure 1. Cells from NSG mice do not express human CD147 protein. (A and B) Gating strategy to identify ACH2 cells (A) and J-Lat 10.6 cells (B) upon staining with CD147 *in vitro*. (C) Representative flow cytometry plots show staining of single cell suspension from diverse tissues obtained from control NSG mice.

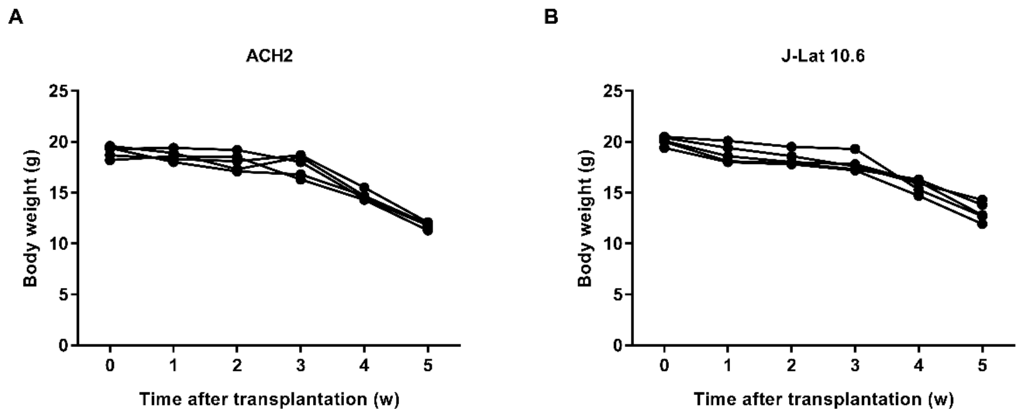

Supplementary Figure 2. Body weight of human cell engrafted NSG mice. NSG mice were engrafted with ACH2 cells (A) or J-Lat 10.6 cells (B). Body weight was monitored every week. N=5.
